# Postural balance during standing at varying virtual heights in children with and without cerebral palsy: a prospective case-control study

**DOI:** 10.1007/s10055-026-01397-0

**Published:** 2026-05-27

**Authors:** Regine Lohss, Morgan Sangeux, Rebecca Winter, Rosa M. S. Visscher, Lilly Kilchmann, Philippe Cattin, Elke Viehweger

**Affiliations:** 1 Centre for Movement Analysis, University Children’s Hospital Basel, Basel, Switzerland; 2 Department of Biomedical Engineering, University of Basel, Hegenheimermattweg 167c, 4123 Allschwil, Switzerland; 3 Institute for Biomechanics, ETH Zurich, Zurich, Switzerland; 4 Department of Orthopedics, University Children’s Hospital Basel, Basel, Switzerland

**Keywords:** Cerebral palsy, Postural balance, Fear of falling, Motion capturing, Virtual reality, Virtual heights

## Abstract

This prospective case-control study explores the possibility to use virtual height exposure for postural balance studies. The study aimed to investigate the effect of standing in a virtual environment on a plank at ground level (VR0) compared to 10 m above virtual ground (VR10) on postural balance. Included were 22 children with cerebral palsy (CP) (age range 8–16 years; male/female: 13/9) and 24 children with typical development (TD) (age range 8–18 years; male/female: 10/14). Participants were instructed to stand still for 40s on two force plates while wearing a head-mounted display, which delivered the virtual environment. Postural balance was quantified via (1) the 95% prediction ellipse area, (2) the average centre of pressure (CoP) velocity, and (3) the CoP amplitude in anteroposterior and mediolateral direction. Additionally, self-reported instability and fear of falling was assessed using a 11-point numeric rating scale. Children with CP tended to exhibit increased median ellipse area, median anteroposterior amplitude, and median velocity from VR0 to VR10 and compared to children with TD, although differences were not statistically significant. Furthermore, median values of self-reported instability and fear of falling increased from VR0 to VR10 in both groups. Our findings suggest that virtual heights may induce changes in postural balance. Therefore, virtual height exposure coupled with force plate measurements may be a promising tool to quantify postural balance.

## Introduction

In children with cerebral palsy (CP), gait disorders are commonly associated with deficits in postural balance (Rose et al. 2002). These are primarily caused by sensory impairments, muscle abnormalities, or lack of motor control (Woollacott and Shumway-Cook 2005). Impaired postural balance affects children’s core stability, thus reducing the capacity to walk efficiently and participate in daily activities (Brogren et al. 2001; van der Heide et al. 2004; Pavão et al. 2014). The Gross Motor Function Classification System (GMFCS) is a widely used system to classify gross motor function of children with CP (Palisano et al. 2008). Postural balance has been shown to progressively decline from GMFCS level I to II and III (Yi et al. 2012).

Postural balance refers to the capacity to maintain the centre of mass within the base of support to prevent falling (Bell 1998). It encompasses any static or dynamic situation and requires strategies to maintain, achieve or restore balance (Pollock et al. 2000). These strategies can either be reactive, responding to unexpected disturbances, or anticipatory, preparing for expected disturbances (Maki and McIlroy 1997). In children with typical development (TD), these postural mechanisms are continuously refined from infancy onward (Brenière and Bril 1998; Assaiante et al. 2005; de Graaf-Peters et al. 2007). Notably, during the initial weeks of walking, head and trunk movements decreased significantly, indicating substantial improvement in trunk stabilization (Ledebt and Bril 2000). However, children with CP show significant delay in postural adjustments such as anticipatory trunk stabilization prior movement execution, reactive responses to external perturbations or compensatory strategies (van der Heide et al. 2004; Carlberg and Hadders-Algra 2005; de Graaf-Peters et al. 2007).

The position of the centre of pressure (CoP), measured via force plates, reflects the neuromuscular response to movements of the centre of mass (Richmond et al. 2021). Parameters such as CoP amplitude, CoP velocity and the 95% prediction ellipse area—derived from the position of the CoP trajectory - are commonly used to quantify standing balance (Quijoux et al. 2021). Although standing balance is closely associated with walking function in CP (Liao et al. 1997; Rose et al. 2002), it is not measured routinely in clinics.

In children with CP, postural balance is widely assessed using a variety of valid methods, each targeting different aspects of postural balance (Pavão et al. 2013). The majority of studies included in Pavão et al. (2013) investigated the effect of visual manipulation on balance during quiet standing, particularly under conditions with eyes open or closed (Rose et al. 2002; Ferdjallah et al. 2002; Donker et al. 2008; Saxena et al. 2014; Pierret et al. 2021). Children with CP exhibited a larger postural sway during standing quietly with eyes opened and eyes closed compared to children with TD (Donker et al. 2008; Reynolds 2010; Saxena et al. 2014; Pierret et al. 2021). Visual feedback of the CoP trajectories decreased sway, less pronounced in children with CP compared to TD (Donker et al. 2008).

Maintaining balance while standing becomes even more challenging when adjusting to different environments. Virtual reality (VR) using a head-mounted display (HMD) allows to fully immerse participants to various virtual environments (VE) within a safe setting. One specific VR application involves simulating heights to better understand the impact of fear-related factors on postural balance (Bzdúšková et al. 2022). It has been shown, that fearful participants demonstrated increased amplitude of CoP displacements (Davis et al. 2009) and increased velocity of CoP displacements (Bzdúšková et al. 2022) with increasing heights, while non-fearful participants decreased CoP displacements (Davis et al. 2009). The effectiveness of virtual height simulation in representing real-world scenarios has been proven by a study showing comparable postural balance adjustments under both real and virtual height conditions (Cleworth et al. 2012). Another study revealed that VR exposure itself, regardless of virtual heights, could impact postural balance, potentially due to visual sensory conflicts induced by the VR exposure (Chander et al. 2021). Adaptations in psychophysiological outcomes such as perceived distress, heartrate (Bzdúšková et al. 2022), and electrodermal activity (Cleworth et al. 2012; Bzdúšková et al. 2022) have also been reported.

These studies have shown that virtual heights, similar to real heights, affect balance during standing in healthy participants. However, there is a lack of studies exploring how children with cerebral palsy adapt their posture in response to changing environmental demands. This knowledge could support the development of more targeted interventions aimed at improving postural balance in real-world settings. Therefore, this study aimed to investigate the effect of standing in a VE at ground level (VR0) and at 10-meter virtual height (VR10) on static stability measures. We hypothesized that (1) both children with CP and those with TD are less stable from VR0 to VR10, and (2) children with CP are less stable when standing in VR compared to those with TD. Reduced stability was expected to be reflected by increases in CoP amplitude, CoP velocity, and the 95% prediction ellipse area.

## Methods

### Study design and participants

Between August 2021 and September 2023, a convenience sample of 22 participants with CP (13 boys, 9 girls) and 24 with TD (10 boys, 14 girls) was recruited prospectively. Participants with CP were recruited through a Children’s University Hospital and diagnosed with spastic CP, 18 unilaterally and 4 bilaterally affected. Twenty participants were classified level I on the GMFCS, and 2 level II. Participants with TD were age-matched to those with CP and were recruited from the local community.

All participants had German language skills and normal or corrected visual acuity.

Excluded were participants with orthopaedic surgeries in the lower extremities (< 12months), botulinum toxin A treatment (< 6months), psychological impairments that would interfere with the study procedure, and with experience in using VR regularly.

This study was registered on clinicaltrials.gov (NCT04879199). The Ethics Commission of Northwestern and Central Switzerland approved the study (2021 − 00435). Eligible participants and their parents were informed prior to the study and provided written informed consent.

### Virtual reality system and environment

The immersive VE was developed via the game engine Unity (Unity Technologies, San Francisco, US, Version 2021.2.8f1). We used the Oculus Quest 2 (Reality Labs, US), a standalone HMD with six degrees of freedom inside-out tracking via four integrated cameras and adjustable lens distance. The HMD has a resolution rate of 1832 × 1920 pixels per eye and a tracking frequency of 60 Hz. The VR application was running on the HMD itself, while a networking layer was used to connect the HMD application to a customized PC application. This PC application was mainly used to control the settings and to change between heights. When changing between heights, the HMD display faded to black for half a second and slowly faded back to avoid sensory conflicts.

The PC was equipped with a 3.60 GHz CPU, 32.0 GB of RAM, a 5 GB GPU and 3.22 TB of storage, ensuring smooth running of the VR application.

**Fig. 1:**
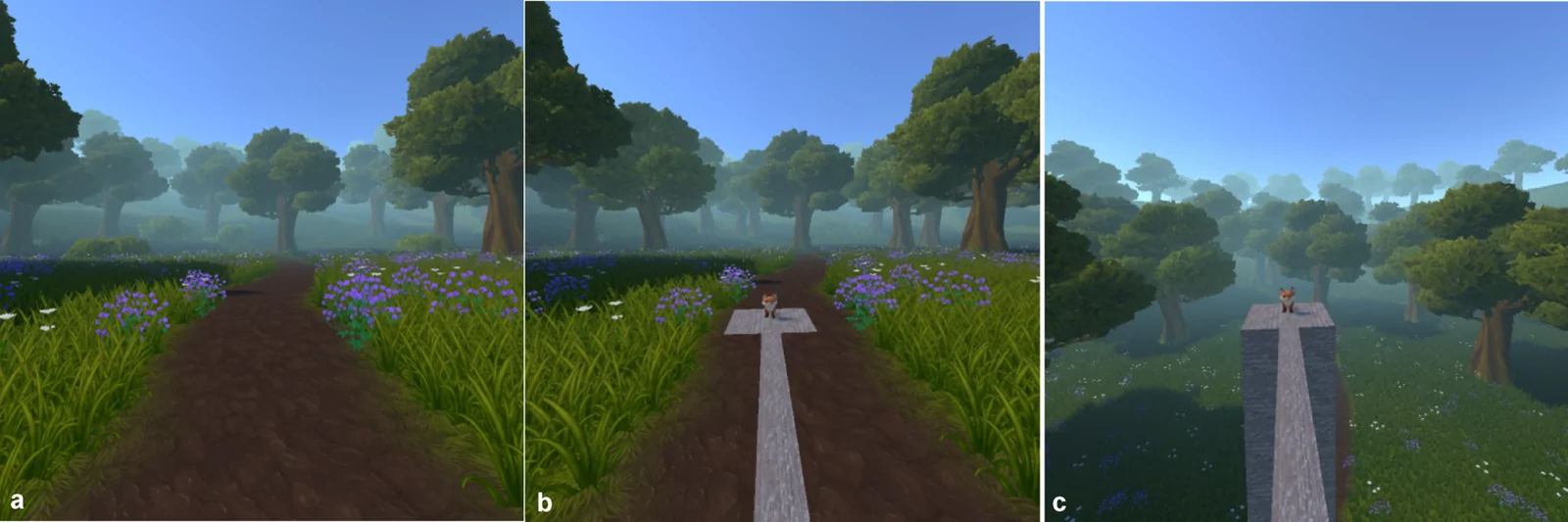
Habituation to the virtual environment (**a**), standing on a plank at ground level (**b**), and at 10 m above virtual ground (**c**). A virtual fox served as visual target during measurements

The VE resembled a forest environment with a plank along a path, spanned between two square platforms, and surrounded by trees (Fig. 1). Length, width and thickness of the plank were set to 7 m, 0.3 m and 0.03 m, respectively. A fox was positioned on the platform in front of the participant and served as a visual target during measurements (Riach and Starkes 1989).

### Procedures

Measurements were performed in a conventional clinical gait laboratory. All participants had reflective markers placed on their bodies. A 12-camera Vicon Motion Capture System (Vicon Inc., Oxford, UK) was used to collect kinematic data during gait as part of the broader study. However, the analysis presented in this paper focuses solely on stability during standing, for which no Vicon data were required or analyzed. The following barefoot conditions were performed:

1. *Habituation to the VE*: Participants were instructed to stand upright, with each foot placed on one force plate, and with their arms hanging relaxed at the sides. The exact alignment and spacing of the feet were not specified but were required to remain consistent across the conditions. The participants put on the HMD for 1 min, while standing in the VE on a path and looking around (Fig. 1a). No data was collected during this condition.
2. *Standing in the VE*: The participants kept the HMD and the posture. Additionally, they were instructed to “stand as quiet as possible” in the middle of the virtual plank at ground level (VR0) (Fig. 1b) and at a 10-meter height (VR10) (Fig. 1c), looking at the virtual fox on the platform ahead. The order of heights was randomly selected for each participant to minimize order effects; however, full counterbalancing was not applied. There was no rest period between the two heights. For each height, two consecutive 40 s trials were recorded. Participants were instructed to wear the HMD for the duration of the entire measurement session.

### Fidgeting review

**Fig. 2:**
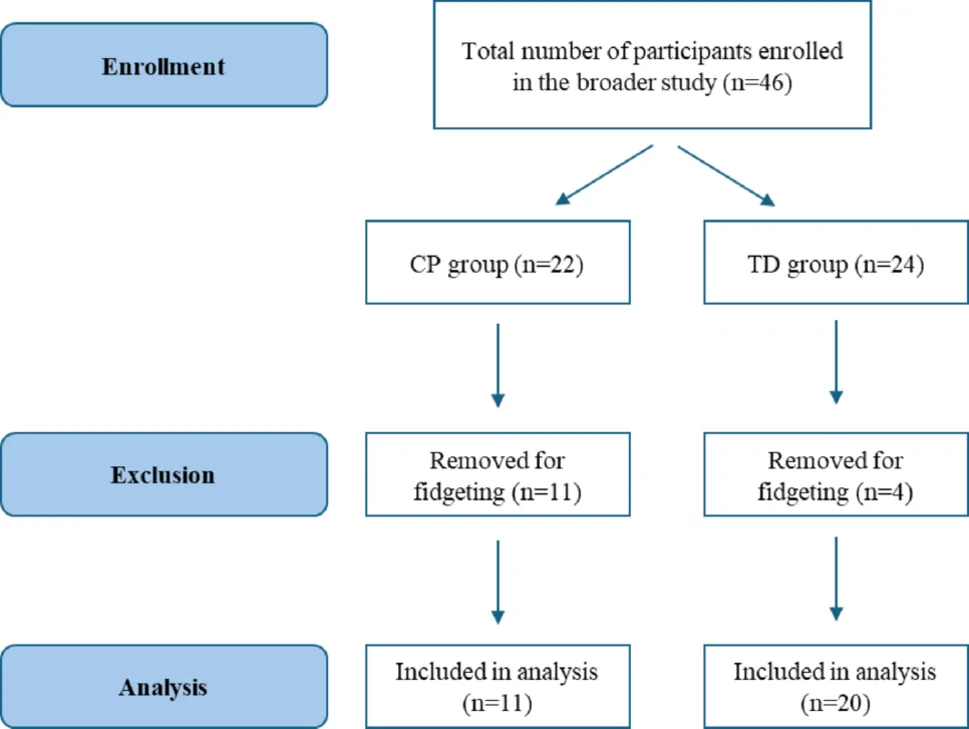
Flowchart of the participants’ recruitment and analysis process. CP: cerebral palsy, TD: typical development

All participants’ motion capture recordings were visually evaluated by RL and LK to identify the presence of fidgeting movements (Tracy et al. 2021). Fidgeting was defined as movements of the head, shoulders, arms, hands, torso, legs, or feet that were clearly intentional and deviated from maintaining a quiet standing posture (Tracy et al. 2021). Cohen’s Kappa was calculated to measure the overall agreement between the two reviewers (Landis and Koch 1977). We included trials for which either neither or only one of the two reviewers identified fidgeting. After review, 11 (out of 22) participants with CP and 20 (out of 24) with TD were included in the analysis (Fig. 2).

### Outcome parameters

The CoP coordinates (x/y) for anteroposterior (AP) and mediolateral (ML) directions were derived for each limb separately using two force plates (Kistler Instrumente AG, Winterthur, Switzerland) with a sampling rate of 1500 Hz. The raw data was exported to MATLAB (Version R2021a, The MathWorks Inc., USA), and passed through a fourth order Butterworth low pass filter with a cut-off frequency of 13 Hz. This frequency was chosen to eliminate high-frequency noise while retaining the relevant frequency components for CoP measurements (Clark et al. 2010; Koltermann et al. 2023). The combined overall CoP was calculated using the weighted average of the vertical ground reaction forces captured by each force plate. The first and last 10 s of data were cut off, considering the middle 20 s of data for analysis. For the combined overall CoP, the following outcome parameters were calculated:

1. Amplitude of the CoP [mm], defined as the distance between the most extreme positions of the CoP throughout data capture in the AP and ML directions.
2. Mean velocity of the CoP [mm/s], defined as the CoP path length divided by the duration of the recording.
3. 95% prediction ellipse area [mm^2^], enclosing around 95% of the CoP data points (calculation according to (Schubert and Kirchner 2014; Duarte 2015).

Additionally, we assessed self-reported instability (“How stable did you feel while standing on the plank?”) and fear of falling (“How strong was your fear of falling off the plank?”) immediately after the first trial of each height using a numeric rating scale ranging from 0 to 10, with higher values indicating higher levels of instability or fear of falling.

### Statistical analysis

The mean for each outcome parameter was calculated before proceeding with the analysis if both consecutive trials of one condition could be included for analysis. Due to the non-linearly distributed outcome parameters (Shapiro-Wilk test), the Wilcoxon Signed-Rank Test was used for within-group comparisons (VR0 vs. VR10) and the Mann-Whitney U Test for between-group comparisons (CP vs. TD). Median values and interquartile ranges (IQR) were reported for the outcome variables, while mean (SD) were reported for the demographic data. The data distribution within each group and condition was visually displayed using boxplots with jittered data points.

Post-processing of the data and calculation of the CoP parameters were performed in Matlab (Version R2021a, The MathWorks Inc., USA). The statistical analyses were performed in R (RStudio, Boston, USA, Version 2023.12.1). Significance level was set to α = 0.05.

## Results

### Agreement fidgeting review

In 15 participants (4 CP, 11 TD) both reviewers identified no fidgeting in at least one trial of both VR conditions. In 16 participants (7 CP, 9 TD) only one reviewer identified no fidgeting in at least one trial of both VR conditions. Cohen’s Kappa was 0.74, indicating a substantial agreement between the two reviewers.

Full demographic details of both groups are presented in Table 1.

**Table 1:** Participants demographics. Group differences between CP and TD were assessed using independent t-tests for continuous variables and chi-square tests for categorical variables

	Group	*N*	Sex M/F	Age (years) mean [SD]	Height (cm) mean [SD]	Weight (kg) mean [SD]	BMI (kg/m^2^) mean [SD]	Leg length (cm) mean [SD]	Affected part(s) unilateral/bilateral	GMFCS level I/II
All participants	CP	22	13/9	11.2 [2.2]	148.0 [11.3]	40.0 [10.8]	18.0 [3.0]	76.6 [6.6]	18/4	20/2
	TD	24	10/14	11.9 [2.9]	154.2 [14.4]	42.7 [12.4]	17.6 [2.4]	81.5 [9.0]	NA	NA
Included in analysis	CP	11	6/5	11.9 [2.3]	153.5 [10.3]	45.0 [12.0]	18.9 [3.7]	79.8 [5.6]	9/2	10/1
	TD	20	6/14	12.4 [2.9]	155.7 [14.3]	43.9 [12.8]	17.7 [2.6]	82.7 [8.9]	NA	NA
	*p*-value		0.18	0.63	0.66	0.82	0.30	0.34		

*CP* Cerebral palsy,* TD* Typical development,* GMFCS* Gross motor function classification system,* M* Male,* F* Female,* NA* Not applicable

### CoP parameters

**Fig. 3:**
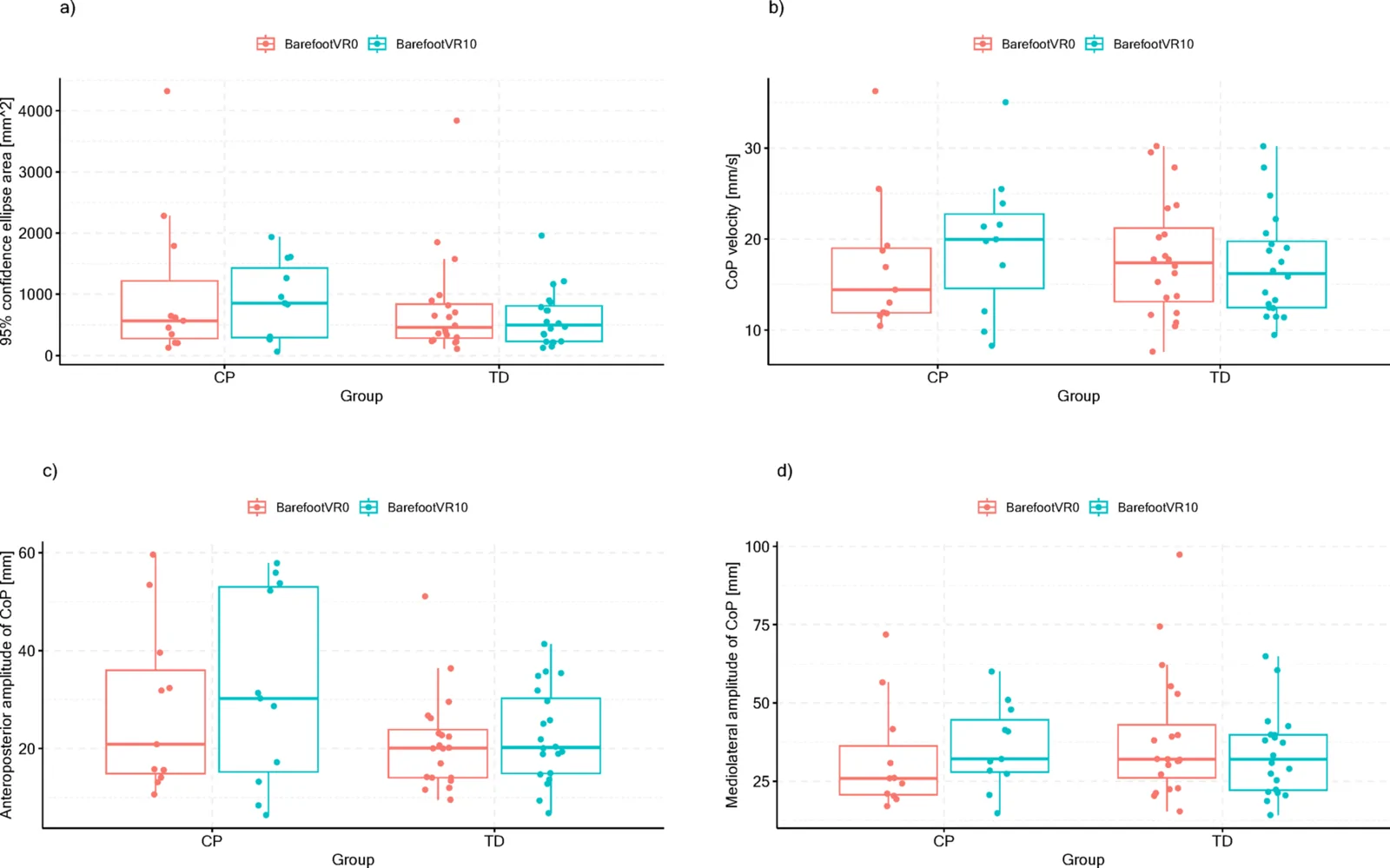
CoP 95% ellipse area (**a**), CoP velocity (**b**), and CoP amplitude in anteroposterior (**c**) and mediolateral (**d**) directions among groups and conditions. CoP: centre of pressure, CP: cerebral palsy, TD: typical development, VR0: standing on a virtual plank at ground level (0 m), VR10: standing on a virtual plank at 10 m above virtual ground

Figure 3 shows the differences in the CoP parameters among groups and conditions. Overall, children with CP tended to exhibit greater postural sway at VR10 compared to both their performance at VR0 and to children with TD. However, no statistically significant differences were found neither for the between-group nor the between-condition comparisons.

In children with CP, there was a trend toward an increase in median ellipse area from 567.0 mm^2^ (IQR 278.9–1218.7) at VR0 to 856.4 mm^2^ (IQR 293.45–1431.2) at VR10 (*p* = 0.97), in median CoP velocity from 14.4 mm/s (IQR 11.9–19.0) at VR0 to 20.0 mm/s (IQR 14.6–22.7) at VR10 (*p* = 0.28), and in median AP amplitude from 20.9 mm (IQR 14.8–36.0) at VR0 to 30.2 mm (IQR 15.2–53.0) at VR10 (*p* = 0.70), although these changes did not reach statistical significance. At VR10, there was a trend toward an increase in median ellipse area from 497.0 mm^2^ (IQR 230.2–810.8) in TD to 856.4 mm^2^ (IQR 293.45–1431.2) in CP (*p* = 0.21), in median CoP velocity from 16.2 mm/s (IQR 12.5–19.7) in TD to 20.0 mm/s (IQR 14.6–22.7) in CP (*p* = 0.36), and in median AP amplitude from 20.2 mm (IQR 14.9–30.2) in TD to 30.2 mm (IQR 15.2–53.0) in CP (*p* = 0.34). However, these differences were again not statistically significant.

### Self-reported instability and fear of falling

**Fig. 4:**
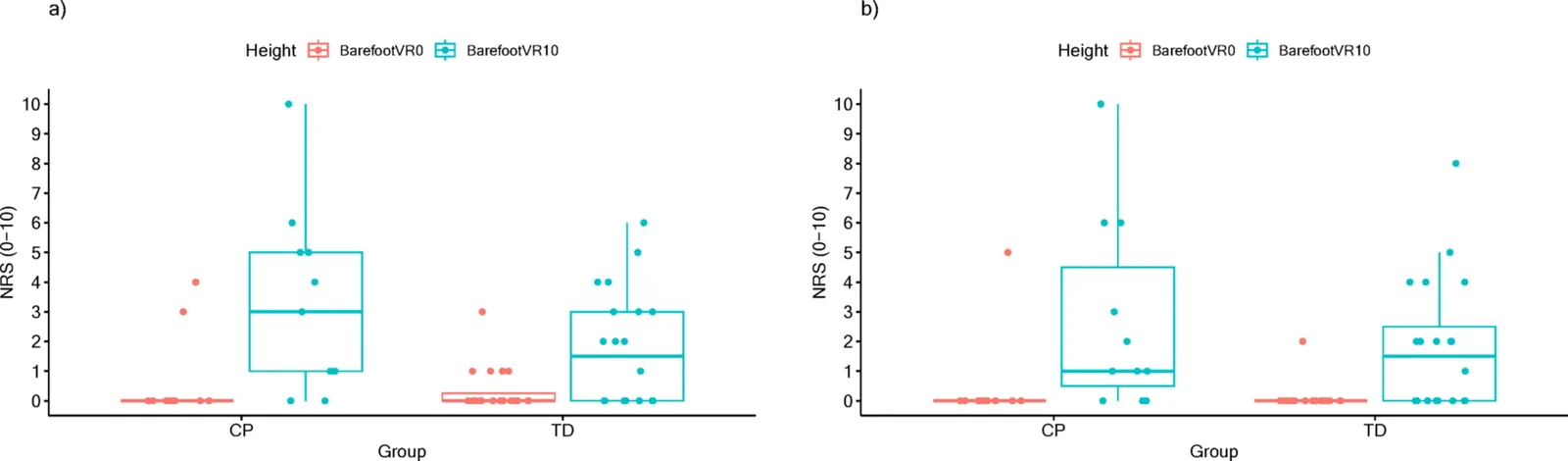
Self-reported instability (**a**) and fear of falling (**b**), measured with a numeric rating scale ranging from 0–10, among groups and conditions. CP: cerebral palsy, TD: typical development, NRS: numeric rating scale, VR0: standing on a virtual plank at ground level (0 m), VR10: standing on a virtual plank at virtual above ground

Figure 4 highlights the differences in self-reported instability and fear of falling among groups and conditions. In children with CP, median self-reported instability on the numeric rating scale increased from 0 (IQR 0–0) at VR0 to 3 (IQR 1–5) at VR10 (*p* = 0.006), and median self-reported fear of falling increased from 0 (IQR 0–0) at VR0 to 1 (IQR 0.5–4.5) at VR10 (*p* = 0.006). In children with TD, only median self-reported fear of falling increased from 0 (IQR 0–0) at VR0 to 1.5 (IQR 0–2.5) at VR10 (*p* < 0.001) (Fig. 4).

## Discussion

The purpose of this study was to explore the impact of a VE at ground level and at 10-meter height on static standing measures in children with CP compared to those with TD. We hypothesized that (1) both children with CP and TD are less stable from VR0 to VR10, and that (2) children with CP are less stable when standing in VR, compared to those with TD. Both hypotheses could not be confirmed. Children with CP showed reactions similar to those of children with TD. However, the findings indicated tendencies that are explained in the following.

From VR0 to VR10 and from the CP to the TD group, the median values of ellipse area, CoP AP amplitude, and CoP velocity increased, though these changes did not reach statistical significance. Although these parameters primarily reflect postural steadiness during quiet standing, the observed changes may also translate to greater sway. Our findings align well with previous studies (Chander et al. 2021; Shim et al. 2022; Szopa and Domagalska-Szopa 2024; Malone et al. 2023). A study revealed comparable changes in CoP-based sway measures from standing in a VE at ground level to standing at 40 m virtual height, albeit in healthy individuals (Chander et al. 2021). Regarding the CoP excursions, children with CP increased median sway from VR0 to VR10 mainly in AP direction. This finding has been confirmed by another study (Shim et al. 2022), arguing that standing balance is maintained mainly due to ankle plantarflexion and dorsiflexion control. The authors concluded that the CoP displacement in AP direction may be more sensitive in this specific context than displacements in ML directions. In our study, the narrow plank and the feeling of falling off the plank might additionally have limited sway in ML directions. Standing at 10 m above virtual ground might have imposed a greater challenge for children with CP to maintain postural steadiness, potentially due to difficulties in adjusting balance strategies to environmental changes (de Graaf-Peters et al. 2007). This originates from impairments in visual, vestibular, and proprioceptive mechanisms, which all contribute to postural control. Exposure to virtual heights primarily challenges the visual system, but the potential underlying mechanism was not addressed. Due to these impairments, compensatory, less efficient, balance strategies such as higher sway are required. Therefore, changes in CoP parameters provide clinically meaningful insights into balance impairments in CP and strategies to compensate for these, and support clinicians to develop treatments strategies and reduce fall risk. Further, greater CoP sway and amplitude are often associated with increased balance difficulties during walking in children with CP (Lohss et al. 2024; Lohss et al. 2025). Children with TD almost showed no changes in CoP-parameters from VR0 to VR10. It could be that the height difference was too small to induce any challenge for maintaining postural steadiness to them. However, it could also mean that they were able to voluntarily control sway, which has already been observed in healthy individuals (Reynolds 2010). According to this study, the ability to control sway even increases as the perceived difficulty of the condition decreases.

The participants’ ratings on self-reported instability and fear of falling provide an indication of how immersive the VE was perceived, or with other words, to which degree they felt being present in the VE. In both groups, median values for instability and fear of falling increased from VR0 to VR10, which demonstrates the height difference was perceived by both groups. The most remarkable difference was found for the CP group, rating self-reported instability from a median of 0 at VR0 to a median of 3 at VR10. Self-reported instability seemed to be higher affected compared to fear of falling. This is not surprising since the awareness of standing on flat ground, despite the VR immersion, might not have evoked the sensation of falling. The overall low levels of self-reported instability and fear of falling in the TD group suggest that neither condition was perceived as particularly challenging for them. Coming back to the ability to voluntarily control, this might have led to a higher proportion of controlling sway even at VR10, at least in the TD group. Overall, median values were small for both ratings, but individual responses varied considerably within groups, most probably due to the small sample size.

To our knowledge, this is the first study investigating the effect of virtual height exposure on standing stability in children with CP. VR is a useful tool to simulate scenarios that are difficult or risky to implement in the real world. We included a relatively homogeneous sample of children with CP in terms of functional skills, with ten participants classified as GMFCS level I and one as level II. However, postural balance may still be variable among participants and cannot be excluded as a potential confounder. We specifically recruited participants without prior exposure to VR to increase the sense of presence in the VR environment. This meant a lot of excitement for the young participants in this study. Unfortunately, this excitement was difficult to contain with the standard instructions given for stability measures leading to the exclusion of many participants after reviewing for fidgeting movement during the quiet standing data collection. The exclusion of participants with fidgeting movements left a smaller sample size for the analysis, leading to reduced statistical power. Further, participants with CP showed a grater variability in self-reported symptoms related to VR exposure, which may be due to a higher heterogeneity in visual, vestibular, or proprioceptive impairments. However, fear of falling and perceived instability were overall low in both groups, potentially due to their awareness of standing on a safe physical surface despite the VR exposure, or the relatively low level of virtual heights. The low level of perceived challenges may have attenuated the observed changes in CoP parameters.

There are other limitations to consider when interpreting the results. First, we measured neither the degree of presence perceived by the participants in the virtual environment nor their perception of height. Only the assessment of self-reported fear of falling provided an indication of how the height difference was perceived. Also, we did not capture additional standing trials outside of the VE. Therefore, we cannot conclude whether there the observed changes were attributed to the virtual heights or the virtual environment itself. Nevertheless, another study reported similar changes in postural balance when comparing virtual to real heights (Cleworth et al. 2012). We also cannot conclude whether there was an effect of the weight of the HMD on balance during standing, which was found by other studies (Horlings et al. 2009; Robert et al. 2016; Almajid et al. 2021). Finally, we did not relate the CoP excursion to the base of support area, which has been proposed by other studies (Popovic et al. 2000; Hof et al. 2005).

## Conclusion

While the data indicated tendencies toward an increase in median ellipse area, CoP velocity and AP amplitude in children with CP from VR0 to VR10, these results did not reach statistical significance, likely due to the exploratory study design and the variability in postural responses among participants. Currently, VR is not yet used as an established assessment tool in populations with higher risks of falls. However, our findings suggest that VR may challenge specific variables of postural control in a controlled and safe setting. To implement VR as a tool in standardized clinical gait laboratory measurements, further validation studies including larger samples are necessary. Furthermore, instructions should specifically be designed to ensure appropriate data collection in young participants immersed in VR, before VR can be considered as a promising assessment tool in populations with higher risks of falls.

## Data Availability

The data that support the findings of this study are available from the corresponding author, R.L., upon reasonable request.
